# Street Collections

**Published:** 1896-04-11

**Authors:** 


### STREET COLLECTIONS.

The Leeds "Hospital Magazine" says: "We hear that the Leeds Ladies' Hospital Committee have been considering the advisability of trying a Hospital Saturday Collection. If the Ladies' Committee would take the advice of the Workpeople's Committee they would soon drop the idea, which years ago was fully discussed by working men, and abandoned as being bad, erratic, and demoralising."

